# Joint controllers in large research consortia: a funnel model to distinguish controllers in the sense of the GDPR from other partners in the consortium

**DOI:** 10.12688/openreseurope.14825.2

**Published:** 2024-02-09

**Authors:** Evert-Ben Van Veen, Martin Boeckhout, Irene Schlünder, Jan Willem Boiten, Vasco Dias

**Affiliations:** 1 MLC Foundation (MLCF), Den Haag, The Netherlands; 2 TMF, Berlin, Germany; 3 Lygature, Utrecht, The Netherlands; 4 InescTec, Porto, Portugal

**Keywords:** data protection, controller, consortium, GDPR, funnel model

## Abstract

Large European research consortia in the health sciences face challenges regarding the governance of personal data collected, generated and/or shared during their collective research. A controller in the sense of the GDPR is the entity which decides about purposes and means of the data processing. Case law of the Court of Justice of the European Union (CJEU) and Guidelines of the European Data Protection Board (EDPB) indicate that all partners in the consortium would be joint controllers. This paper summarises the case law, the Guidelines and literature on joint controllership, gives a brief account of a webinar organised on the issue by Lygature and the MLC Foundation. Participants at the webinar agreed in large majority that it would be extreme if all partners in the consortium would become joint controllers. There was less agreement how to disentangle partners who are controllers of a study from those who are not. In order to disentangle responsibilities, we propose a funnel model with consecutive steps acting as sieves in the funnel. It differentiates between two types of partners: all partners who are involved in shaping the project as a whole versus those specific partners who are more closely involved in a sub-study following from the DoA ^i^ or the use of the data Platform. If the role of the partner would be comparable to that of an outside advisor, that partner would not be a data controller even though the partner is part of the consortium. We propose further nuances for the disentanglement which takes place in various steps.

Uncertainty about formal controllership under the GDPR can stifle collaboration in consortia due to concerns over (shared) responsibility and liability. Data subjects’ ability to exercise their right can also be affected by this. The funnel model proposes a way out of this conundrum.

## Abbreviations and acronyms

**Table:** 

Abbreviation/ Acronym	DEFINITION
CJEU	Court of Justice of the European Union
DAC	Data access committee
DoA	Description of Action
EDPB	European Data Protection Board
GDPR	General Data Protection Regulation
IMI	Innovative Medicines Initiative
MLCF	MLC Foundation

## Disclaimer

The views expressed in this article are those of the author(s). Publication in Open Research Europe does not imply endorsement of the European Commission.

## Introduction

The General Data Protection Regulation (GDPR) ^1^ states the generic rules to which all data processing should comply either which takes place in Europe or which is targeted at European citizens. GDPR compliance starts with entities responsible for compliance, in GDPR terms the “controllers”. Controllers decide on the purposes and the (essential) means of data processing (article 4, GDPR) ^2^. A few provisions also refer to responsibilities of the processor in the sense of articles 28.3 under h and 28.4 GDPR, but most of the responsibilities of the processor are a derivative of those of the controller. Noncompliance can have far-reaching consequences, ranging from high fines by the regulatory authorities (chapter VIII, GDPR) ^2^ to civil liability according to national law.

If a party is neither a controller nor a processor, it does not have responsibilities under the GDPR. 

Decisions of the European Court of Justice in 2018 and 2019 broadened the concept of joint controllers, see section 5.3 infra. The EDPB followed up on this in its guidelines on controllers and processors, first in the draft Guidelines of September 2020 and then the final guidelines of July 2021 ^2^. The case law and the (draft) Guidelines have raised the question of who would be controllers in large research consortia. Especially in the life sciences, the European Union encourages such large consortia either under the umbrella of Horizon 2020 or the Innovative Medicines Initiative (IMI), now the Innovative Health Initiative (IHI) ^3^. As it is about life sciences, many sensitive data will be processed in such research. If taken to the extreme, all partners in the consortium would be joint controllers. This can be problematic for individual consortium partners: they could be held responsible for data processing within the consortium, while their actual influence on the data processing is modest or very indirect. In practice the actual data processing is usually performed by a specific subset of consortium partners. Uncertainty about formal controllership under the GDPR can stifle collaboration in consortia due to concerns over (shared) responsibility and liability. Data subjects’ ability to exercise their rights vis-à-vis data controllers can also be affected by this. It should be made transparent to the data subjects who are responsible for data processing, but it is questionable whether a very long and indiscriminate list of consortium members would clarify anything to them (article 26, GDPR) ^2^. In effect, as we will argue, it seems more likely that overly broad interpretations would prove counterproductive, weakening, instead of promoting, data subjects’ empowerment and trust.

In the following, drawing on our experiences in participating and advising large research consortia in the health and (bio)medical sciences, we will further discuss the issue of (potential) joint controllership in this context. The discussion is partially based on a webinar jointly organised by MLCF and Lygature in April 2021. We propose a ‘funnel-and-sieves’ model for deciding who are joint controllers for the data processing in research consortia and who are not. Such a discussion should build on the case law, the Guidelines and literature on joint controllership as will be discussed below.

Large EU-funded research consortia form the starting point of our analysis. These have a grant agreement and consortium agreement which must meet certain criteria and has narrowly defined or perhaps one should say, rigidly defined, responsibilities of the partners vis-à-vis each other and the funding agency as described in the Description of Action (DoA). There is no choice here if one wants to receive the funding. Other situations of research cooperation were out of scope

## Basics about GDPR controllership 

### The provisions in the GDPR

The following provisions matter most of all here.

GDPR Article 4(7), on the definition of a controller:

“‘controller’ means the natural or legal person, public authority, agency or other body which, alone or jointly with others, determines the purposes and means of the processing of personal data; where the purposes and means of such processing are determined by Union or Member State law, the controller or the specific criteria for its nomination may be provided for by Union or Member State law”;

Article 26, on Joint controllers:

1. “Where two or more controllers jointly determine the purposes and means of processing, they shall be joint controllers. They shall in a transparent manner determine their respective responsibilities for compliance with the obligations under this Regulation, in particular as regards the exercising of the rights of the data subject and their respective duties to provide the information referred to in Articles 13 and 14, by means of an arrangement between them unless, and in so far as, the respective responsibilities of the controllers are determined by Union or Member State law to which the controllers are subject. The arrangement may designate a contact point for data subjects.
2. The arrangement referred to in paragraph 1 shall duly reflect the respective roles and relationships of the joint controllers vis-à-vis the data subjects. The essence of the arrangement shall be made available to the data subject.
3. Irrespective of the terms of the arrangement referred to in paragraph 1, the data subject may exercise his or her rights under this Regulation in respect of and against each of the controllers.”

### Case law

Three judgements on joint controllers were issued under the former Directive 95/46/EC (the Data Protection Directive, DPD). Legal definitions did not materially change with the GDPR, so previous case law decisions pertaining to controllership under the DPD are still valid today. Under the DPD, the responsibilities resulting from joint controllership were narrowly constructed, while under the GDPR joint controllership may lead to joint and several liability. If anything, the strictly legal questions over determining controllership on which this paper focuses have thereby become even more urgent.

Wirtschaftsakademie Schleswig-Holstein ^4^ was a case where Wirtschaftsakademie offered educational services by means of a fan page hosted on Facebook. Fan pages are user accounts that can be set up on Facebook by individuals or businesses. To do so, the author of the fan page, after registering with Facebook, can use the platform designed by Facebook to introduce himself to the users of that social network and to persons visiting the fan page, and to post any kind of communication in the media and opinion market. Administrators of fan pages can obtain anonymous statistical information on visitors to the fan pages via a function called ‘Facebook Insights’ which Facebook makes available to them free of charge under non-negotiable conditions of use. The Wirtschaftsakademie was charged with infringing German data protection legislation. The company replied that it was not the controller of the personal data processed by the fan page, but that only Facebook is. The Court of Justice of the European Union (CJEU) emphasized the need for a broad interpretation of controllership to ensure a high level of protection for data subjects. It held that there are two controllers in relation to a Facebook fan page. First, Facebook serves as a controller from the perspective of personal data protection, as it primarily determines the purposes and means for users and visitors of fan pages. Secondly, fan page administrators such as the Wirtschaftsakademie serve as controllers as they subscribe to Facebook’s conditions of use. Fan page administrators were considered to determine the purposes (the objective of establishing a fan page) and the means of data processing by defining parameters of data collection, including the target audience. Considering that a fan page administrator influences these modalities (carried out by Facebook), the Wirtschaftsakademie as a fan page administrator was classified by the Court as a controller as well, even though it only received analytics data in anonymized form. The Court there by ruled in favour of a broad interpretation of controllership.

Jehovan Todistajat ^5^ was a Finnish case where individual members of the ‘Jehovah witness community’ took notes of the households which they visited during their door-to-door preaching. The ‘Jehovah witness community’ encouraged door-to-door visits and gave training on how to do this, but would not receive the individual member’s notes. The CJEU ruled that the ‘Jehovah’s Witnesses Community’ should be qualified as a controller in relation to the personal data collected by its members through door-to-door preaching even though it would not obtain that data. Through its verdict, the Court established that controllership of personal data doesn’t presuppose that the controller has access to such data.

In FashionID ^6^, the website of an online shop had a ‘social plugin’ which caused a visitor’s browser to request data from the provider and transmitted data to that provider (the Facebook ‘like’ button). This transmission to Facebook apparently happened even if one did not click on the ‘like button’. Simply visiting the site was enough to send data to Facebook. Unsurprisingly, FashionID was qualified as a joint controller together with Facebook for the data sent to Facebook via its website. The Court recalled the need to broadly interpret the notion of control to ensure a high level of protection for data subjects. The website operator was said to enable Facebook ‘to obtain data of visitors to its website (…) regardless of whether or not the visitor is a member of the social network’. Hence it exerted ‘decisive influence over the collection by Facebook and transmission’ to Facebook’.

On the other hand, however, FashionID was not considered a controller for the subsequent processing of the data by Facebook. The Court thereby established that one is only joint controller for that part of the data processing where purposes and means are inextricably linked.

### The EDPB guidelines

The rules on controllership have subsequently been qualified further by Guidelines established by the EDPB, the joint European body composed of representatives of the EU national data protection authorities as established by the GDPR. The EDPB contributes to the consistent application of data protection rules throughout the European Union, and promotes cooperation between the EU’s data protection authorities, notably through providing guidance (article 70, GDPR) ^2^. Such guidance is followed in particular by the data protection authorities themselves in their supervisory capacity.

Insofar as relevant here the EDPB Guidelines on controllership summarise the criteria for joint controllers as follows: ^2^

“The overarching criterion for joint controllership to exist is the joint participation of two or more entities in the determination of the purposes and means of a processing operation. Joint participation can take the form of a common decision taken by two or more entities or result from converging decisions by two or more entities, where the decisions complement each other and are necessary for the processing to take place in such a manner that they have a tangible impact on the determination of the purposes and means of the processing. An important criterion is that the processing would not be possible without both parties’ participation in the sense that the processing by each party is inseparable, *i.e.* inextricably linked. The joint participation needs to include the determination of purposes on the one hand and the determination of means on the other hand.”

At point 52 the EDPB adds that

“The assessment of joint controllership should be carried out on a factual, rather than a formal, analysis of the actual influence on the purposes and means of the processing”.

In the absence of common or converging decisions, joint controllership may also be attributed indirectly. The EDPB (at point 60):

“In addition, when the entities do not have the same purpose for the processing, joint controllership may also, in light of the CJEU case law, be established when the entities involved pursue purposes which are closely linked or complementary. Such may be the case, for example, when there is a mutual benefit arising from the same processing operation, provided that each of the entities involved participates in the determination of the purposes and means of the relevant processing operation. However, the notion of mutual benefit is not decisive and can only be an indication.”

In this case, in the absence of common or converging decisions, the examples given from the case law point at a common economic interest and linking both interests by a decision of one party to use the means provided by the other party to pursue those interests. But the examples provided by the EDPB are already examples where controllership is ‘inextricably linked’. In our opinion, it therefore remains unclear when that indication of mutual benefit would be so strong that common or converging decisions are not by themselves sufficient already. 

### The situation for large research consortia

The case law of the CJEU only addresses situations in which two parties are involved. Large research consortia have many partners, sometimes over one hundred. These partners will have different roles in the consortium, such as data provider, sponsor of a clinical trial, trial site, data analyst, scientific, ethical or legal counsel, or project management. Sometimes a partner has overlapping roles, as more than one function of the partner is involved in the project. All are committed to the purposes as described in the call and the proposal which led to the funding. The essential means are laid down in the DoA to be further refined during the project. Partners who are also work package leads are usually represented in a ‘management board’ of ‘steering committee’ which approves the major documents, such as a trial protocol or the data management plan. All partners have an interest, including financial, in the project being successful. In that sense, it is difficult to see why any consortium partner should not be qualified as a joint controller for the data processing generated by the project. The fact that many partners do not process personal data from the participants is not an argument as such: as we have seen, one can also be joint controller without having access to the personal data. 

The EDPB also refers to joint controllership in the context of research collaborations. It states that: ^2^

“Several research institutes decide to participate in a specific joint research project and to use to that end the existing platform of one of the institutes involved in the project. Each institute feeds personal data it already holds into the platform for the purpose of the joint research and uses the data provided by others through the platform for carrying out the research. In this case, all institutes qualify as joint controllers for the personal data processing that is done by storing and disclosing information from this platform since they have decided together the purpose of the processing and the means to be used (the existing platform). Each of the institutes however is a separate controller for any other processing that may be carried out outside the platform for their respective purposes.”

However, research consortia have more partners than the research institutions whose researchers perform the actual research involving personal data. What about those? As indicated above, in theory these partners could still meet the criteria for joint controllers.

Additionally, the EDPB example refers to a data platform where all data will be merged and joint research will be performed on all data on that platform. The research reality is often more complex. There can be a platform which is the joint initiative of all partners for certain broadly defined research purposes. However, which research will actually take place on that platform is decided for each sub-study separately by certain partners, but not by all partners together. Would the implementation of such a system already be enough to be considered joint controllers for those entities (partners in the research consortium) which have decided about the broad purposes of the platform and obviously certainly contributed to the means and the conditions for using the platform but did not specifically draft the protocol for the sub-study? 

### Literature

Since the webinar took place, two publications relevantly address aspects of the issue. A publication by Becker *et al.* was specifically dedicated to the issue of joint controllers in research ^7^. It discussed a situation involving two parties, one being a data provider and another using those data for research. The paper outlines the criteria for when, if a data provider makes data available for research (either by transferring data or by allowing access), they become a joint controller for the research performed on that data by the researcher requesting the data. In general, Becker *et al.* stated that one being a data provider is not a sufficient condition to become joint controller with the research institution receiving and analysing the data. Additional criteria must be met, such as that the data provider and recipient researchers have drafted the research plan together or will analyse the research data together and use it as support to publications. Becker *et al.* are in our opinion correct in making those distinctions. However, their framework does not give a straightforward answer for the data processing as initiated and guided by larger research consortia. In that case all activities of the partners are meant to contribute to the results of the consortium, but it might be argued that not all data processing is inextricably linked to the activities of each of the partners. We will come back to that in the discussion. 

From a very different perspective Finck criticises the broad scope of joint controllership in the case law discussed above ^8^. Her point of reference is most of all the individual data subjects or small businesses which are made joint controllers for part of the data processing happening on social media while not being able to effectively exercise control. She combines this with the narrow definition of the ‘household exemption’, as follows from earlier case law of the CJEU ^9^. Just as its predecessor (Directive 95/46/EC) the GDPR does not apply to data processing by a natural person in the course of a purely personal or household activity (article 2.2c, GDPR) ^2^. Processing on social media is not purely personal and neither was that of the Jehovah witnesses, who in little groups of likeminded friends went door to door to spread their faith. Combining the narrow definition of the household exemption with the CJEU case law on joint controllership, Finck submitted that a too-broad notion of controllership would ‘pulverise’ accountability as meant by the GDPR of ‘real’ controllers and has no additional benefit for safeguarding data subjects’ rights and interests. Finck proposed a *de minimis* test: “only parties that determine the purposes and the means beyond the mere choice of a platform or service and the enabling of someone else’s processing should be controllers.” According to Finck, this would be more in line with the rationale of the GDPR. Moreover, this would reflect a more appropriate risk-based approach towards data protection, in which the risks for non-compliance are “imposed on those that have actual decisional power over the processing of personal data and reap the benefits therefrom.”

We agree with Finck that a certain *de minimis* test is an important reference point in the discussion: only parties deciding on the “essential means” of processing, not just any means, should qualify as controllers. In the context of the controller-processor distinction, the EDPB has also stated that the controller should decide about the essential means, but that non-essential means may be decided by the processor alone, or in other words, if an entity decides on non-essential means for data processing that does not as such make that entity a controller ^2^. At the same time, Finck started from a very different context of growing decentralization of data processing operations, where controllers may even be “unaware of their respective existence”. This context cannot be directly transposed to that of large research consortia.

### Expert perspectives on the issue of joint controllership: outcomes of a webinar

On 13 April 2021, MLCF and Lygature organised a webinar on the issue with over 100 expert attendants from various backgrounds (biomedical researchers, legal, management). ^ii^

The authors of this article present their perspectives, followed by an online discussion with the attendants. 

In the first presentation Evert-Ben van Veen summarised the case law and the EDPB position in the then draft guidelines. Translated one-on-one to the situation of research consortia, all partners in a research consortium would become joint controllers. This would take things to the extreme. He proposed certain in- and exclusion criteria, to be discussed in the following section.

After that Jan Willem Boiten gave a general overview of the use cases as observed in large medical research consortia, drawing generic distinctions between how data are exchanged in research consortia and the possible consequences for joint controllership.

Martin Boeckhout then explained the HEAP project, a research platform under construction aimed at facilitating the assessment of the impact of the exposome on human health. The HEAP Platform will contain high-quality exposome data from five different cohort studies, and will be made scalable to any research setting. HEAP has elements of a common data platform (where data are shared) and a federated system. Whether HEAP partners can be considered joint controllers will depend on the governance. Two extreme scenarios can be distinguished: on the one hand a governance where all partners decide which research may be performed on the platform and on the other hand a “governance-lite”, where the platform only provides a service to process data and leaves it to the data provider and researcher to make arrangements for data sharing. In the first case the partners could easily be seen as joint controllers, while in the latter case they would not. Intermediate scenarios can also be envisaged.

Vasco Rosa Dias described the RECAP preterm data platform, a geographically diverse network of national and European cohorts of infants born preterm, constituted over a 30-year time span. This platform is a purely federated system where local nodes’ managers are responsible for harmonising, curating, describing, storing and, with great autonomy, deciding whether to give access to their data and under which conditions. In fact, once deployed, data nodes are managed essentially at the local level, from both a technical and a governance/legal perspective. The RECAP-preterm consortium agreed on ‘terms of use’ to deploy the platform. Though this platform has been established with the involvement of all RECAP preterm partners and all agreed to the terms of use, Vasco argued that joint controllers are only the research entities/researchers requesting data and those data providers that have a role in the research protocol, beyond merely giving access to the data. For the same reason, a partner merely responsible for the design, development and/ or maintenance of the platform, but not involved in the design of any specific project carried out through it, nor providing or requesting data, but still having access to the data (for example via control of the software) could arguably be seen as a data processor (or a service provider) and not a (joint) controller.

Irene Schlünder concluded with discussing the IDEA-FAST platform. IDEA-FAST will conduct two observational studies where the participants use devices which monitor sleep and fatigue. IDEA-FAST has established a data platform where all the study data will be merged. In IDEA-FAST, there is a significant involvement of all partners deciding on data types to be collected, the choice of devices which will provide the data, and the scientific protocol. Yet, some involvement is more intrinsically linked to the data processing than others, such as the study team who finalised the protocol, the clinical centres which recruit the participants, the researchers who will analyse the data and legal and ethical experts advising on the set-up and conduct of studies or about the governance of the data platform. Schlünder highlighted the potential negative consequences of assuming joint controllership too easily. Joint controllership comes with joint responsibilities and liability. Yet, her last slide showed that the criteria for controllers being “inextricably linked”, based on the criteria the EDPB provided, will quickly be met by partners when they have mutually agreed upon actual data processing. 

During the subsequent discussion, almost all participants agreed that research consortia face a problem with respect to the discussed issue. It might even be stated that since funders have a role in deciding the purposes of data processing, funders could, in an extreme interpretation, also be considered controllers – a role which research funders certainly will eschew. The consensus was that a large number of joint controllers does not add any value for the protection to rights and interests of the data subjects/participants in the research of the consortium.

According to the participants, it also creates problems for the consortium. Joint controllership means joint liability in the sense of article 82 in the GDPR, meaning that every party can be held liable for the joint processing even if there is no fault involved of the party which is held liable. The parties concerned can make internal arrangements about who will hold the other party harmless if that party has been held liable (say if data breach occurs at Party 1 and Party 2 is held liable and must pay damages, then Party 1 will indemnify Party 2). Yet, this would make consortium agreements quite complicated.

Participants also considered it crucial that new contractual templates, guidelines and model agreements be devised to deal with these complexities. For instance, although the widely used Desca model consortium agreement for EU-funded research consortia has recently been updated to take the GDPR into account, the generic clauses do not solve the dilemma discussed here ^10^ ^iii^.

The problem will also exist for the sustainability arrangements when the project ends, and the legacy data have to be preserved. Detailed arrangements might be set up about who may use those data under what circumstances. In that case, the entity or entities deciding about whether these criteria are met might be considered joint controllers. 

Some participants were of the opinion that federated solutions, where instead of data transfer only algorithmic access ^11^ ^iv^ is granted and data remains on-premise, could solve many problems. Although complying with data protection requirements may be made easier by such access arrangements in some respects ( *e.g.* data security), federated data architectures do not solve the puzzle about joint controllership. RECAP-preterm is using such a system, but a clear demarcation is still needed between responsibilities for the actual study conducted via the system and responsibilities shared by the assembly of partners making the system possible.

It was mentioned that transparency to the data subjects/participants is key. Via this transparency they can also exercise their rights. This is also highlighted by 26.1 GDPR which states that joint controllers can appoint one contact point. The second section of article 26, however, states that the essence of the agreement between joint controllers should be made available to the data subject. That could be a challenge when the data processing and the division of responsibilities is complex as follows from the DoA and/or the Consortium Agreement between the partners. It is difficult to see how the data subject is truly informed by a summary of the complex interactions between the various Work Packages in a project. The only relevant information for data subjects is whom is actually responsible for their data and whom to contact in case of questions.

Finally, there was some discussion about whether in the case of joint controllers each controller should notify a data breach or if this responsibility can be delegated to one of the (joint) controllers. According to some participants, the Dutch Supervisory Authority seemed to propose the first solution. Yet, the final Guidelines on the concept of controller and processor ^2^ seem to indicate that the agreement between the controllers can appoint the controller who will do so (at point 191) though the Supervisory Authorities are not bound by that agreement (at point 192).

## Towards a clearer demarcation: the funnel model

As was shown in the presentations of Van Veen and Schlünder at the webinar, partners can have different and sometimes overlapping roles in the consortium. The webinar was not conclusive about which criteria should be applied to distinguish between roles that are merely supportive to the project and roles which lead to data processing being inextricably linked and therefore qualifying as joint controllers.

The slides from Van Veen and Schlünder highlighted that in theory, joint controllership exists when all partners jointly collaborate within the project. It is not necessary that one has overall control but, at least, that one step in the data processing cannot be made without the other. In a way, that is the essence of the cooperation in a research consortium. That all partners would be joint controllers, resembles the EDPB criterion that all activities are ‘inextricably linked’ and hence so is the data processing resulting from the activities. However, a large majority of the participants at the webinar considered this conclusion too extreme. We concur, but then the challenge is to find criteria which distinguish partners who are controllers and those who are not.

To find those criteria, we propose to make several relevant distinctions. We propose a funnel with various sieves. Those which remain on a sieve are not controllers, those which pass through could be controllers. At the end of the funnel, the controllers remain.

### First sieve

Although the project proposal and the DoA describe purposes and means in broad lines, these ambitions are refined during the project when performing actual studies involving data processing. Controllers are only those who are in charge of the specific studies which result from the project, or, in the case of a data platform, who control the studies which are performed via that platform.

In both cases the broad ambitions of the consortium are narrowed down via the actual studies, hence the data processing, performed via the means of the consortium. For instance, the design and conduct of studies within a consortium may be led by a particular working group, in which only some of the partners are actively participating.

### Second sieve

Yet, many are involved in this ‘narrowing down’ and we need a second refinement, which, in our opinion, follows from the following distinction. Some partners have a concrete involvement in the actual data processing, while others have a mere auxiliary role. The latter typically provide advice, are involved in the project management, or perform some technical endeavours for the benefit of the partners who ultimately determine the purposes of each study. Though these may be active partners in a project, those services could in theory also have been subcontracted. Those partners who provide services which also could have been subcontracted but for any variety of reasons are performed by consortium members ^v^, are usually not controllers. So, even if that partner strongly influenced or participated in drafting the research protocol by for example giving legal and/or ethical advice, it should not be considered a controller. An external party might have done the same.

### Third sieve

We need to refine further using a third sieve to determine the ‘concrete stake’ as follows:

3a. The partner(s) have drafted the protocol and/or have decided on how to perform the analyses on which the results will be based and/or:
3b. The partner(s) have taken up the overall responsibility for the data platform and decide what analyses may be performed on the platform and under which conditions.

The distinction between 3a and 3b comes from the two distinctive aspects which research consortia can organise: studies and/or a data platform. Some consortia only organise the former. Many do the latter, which will then lead to studies performed on the platform. Yet, the criteria for distinguishing between controllers for studies do not coincide with those who manage the platform. The latter might become controllers as well.

Criterion a reflects the criteria by Becker *et al.* ^7^: only those parties actually involved in deciding which research (purposes) is performed with which data (means) should in principle be considered controllers. A partner providing data, without being involved in the protocol or the analyses, should not be considered a controller of the study data. Of course, the partner will be a controller of the data at the data source and will need a legal basis to be able to share the data for the study. However, that by itself does not make the data source a controller of the study data.

Criterion b reflects the analysis made regarding the HEAP project that data platforms can involve different modes of governance and distributions of power regarding the purposes and means for which the platform is used.

### Fourth sieve

Yet, these criteria are still too broad. Regarding 3a, many partners might influence the protocol or be called to analyse data and there will always be a grey area regarding whether their services could be outsourced or not (second sieve). In the case of 3b we can recall ethics committees. It would be a stretch to argue that committees only involved in vetting and approving others’ research proposals should also be considered joint controllers. Yet for data access committee, the situation might be more complex as will be discussed below.

We therefore need further refinements. Partners who have passed this third sieve are divided. Those of 3a will go through sieve 4a. Partners fit the 3b criteria, will go through sieve 4b.

Sieve 4a would be that they have a preponderant stake in the drafting of the research protocol or analysis plan and/or the conduct and analysis of the research. Obviously, this leaves a grey area. The consortium should decide and find a balance between eschewing responsibilities as a joint controller and offering an understandable privacy statement to the participants which reflects the realities of who, in spite of the involvement of many partners, has had the strongest say in the final protocol or will be the most involved in the data analysis design ( *e.g.* which data are analysed, by whom and for which specific research purposes). Those partners are controllers.

Sieve 4.b focuses on gatekeepers usually referred to as ‘data access committees’ (DAC’s) ^12– 14^. Research data should be made FAIR ^15^, with data generated in research funded through European Commission-based framework programmes being made “as open as possible, as closed as necessary” ^16^. DAC’s ideally have mixed composition of representatives of the consortium, independent advisors and participant representatives. ^vi^ If the role of such a DAC was only to vet whether the research is scientifically sound, can be done with the data, data access is proportional to that purpose, data processing is safe and meets the reasonable expectations of the participants, such a DAC or its members would not become joint controllers. In such a case, the role of the partner representatives in the DAC is comparable to that of members of an independent committee. Such partners can still have an important role in the decision as they know most about the provenance of the data, their re-usability or the functioning of the platform and the research pipelines which can be supported by the platform, while not being considered as joint controllers of the research conducted by others. However, if the DAC went beyond these generally accepted criteria and tried to influence from their own perspective what research may be performed (the purposes) or how it should be performed (the means) then they should be considered joint controllers together with the researchers who proposed a study.

## Concluding remarks

Case law of the CJEU has broadened the concept of controller with the consequence that one can become a joint controller for data processing even without having access to the personal data. This was a potential consequence of the guidelines from the EDPB on the concepts of controller and processor. The main criterion is whether the decision about purposes and essential means of the data processing is ‘inextricably linked’. This poses problems for large research consortia in the biomedical sciences where all work packages of the DoA are ‘inextricably linked’. Without such linking the project wouldn’t have been funded. In a webinar which was organised about this subject, the consensus was that considering all partners, joint controllers would be extreme and detrimental to the transparency towards the participants. Yet, there was less consensus on how the line could be drawn between partners who should be considered (joint) controllers and partners of the consortium who are not.

Hence, we developed the funnel model with four sieves:

- The first sieve distinguishes between the broad ambitions of the project and specific studies involving data processing which result from it. In those studies purposes and means are narrowed down. Possible controllers can only be partners who are concretely involved in those specific studies or what research may be performed on the data platform.
- The second sieve distinguishes between partners who have an auxiliary role in those studies, which in theory often could also have been outsourced and partners who have not such a role and either have drafted the protocol and/or are involved in analysing the data, or partners have taken up overall responsibility for the data platform and decide what analyses may be performed on it. Partners with an auxiliary, advisory role are in principle not controllers.
- Among the partners who have drafted the protocol and/or involved in analysis of the data, a further distinction must be made. Many might be involved in the design, but that role might be limited, such as supporting with the power calculation. The role should be preponderant about purposes and essential means to become a controller. Obviously, the sponsor of a clinical study would qualify as such but there may be others. This may leave a grey area, but we submit that as long as the choice adds more to the transparency towards participants than obfuscating it, such a choice is defensible.
- The responsibility for access and use of a platform is usually delegated to a DAC. The partners involved in any DAC which bases its decisions on clear, objective criteria do not become joint controllers for the studies performed via the platform. However, if the partners via a DAC became involved in the purposes and means of a study, beyond those criteria, they would become joint controllers together with those who initiated the study using the platform.

The proposal here would have clear advantages both for the data subjects and the consortium. For data subjects, the privacy statement (articles 13 and 14, GDPR) ^2^ in the light of article 26.2 GDPR can be comprehensible and will not be obfuscated by listing all partners with complicated division of responsibilities. If the data subject had a complaint, the complaint could be directly addressed to those partners who are really involved in the data processing. The consortium would not need the legal complexities of arrangements about joint controllership of personal data while many of the partners could hardly influence the actual dataflows and security of the data. Establishing such arrangements with recourse to legal departments with usually a huge waiting time, could stifle the whole project, while in fact studies from the project should be performed to generate the results for which the project was funded; obviously in an ethical and data protection compliant way. We submit that using the funnel model as discussed above leads to good data protection compliance and more efficient use of scarce research funds. 

## Data Availability

1) An overview of the slides presented at the webinar on joint controllers in large research consortia on 13th April 2021 can be accessed through the following link: https://mlcf.eu/wp-content/uploads/Program-Webinar-Joint-Controllers-with-links-to-presentations.pdf 2) The Anonymised chat of the webinar can be accessed through the following link: https://mlcf.eu/wp-content/uploads/Chat-webinar-joint-controllers.pdf

## Footnotes

^ii^ Slides and the chatlog can be found respectivelyat https://mlcf.eu/presentations-from-the-webinar-on-joint-controllers-in-large-research-consortia/ and https://mlcf.eu/wp-content/uploads/Chat-webinar-joint-controllers.pdf (last accessed 27-04-2022).

^iii^ Note that the new DESCA model consortium agreement assumes that joint controllership is generally shared only between a subset of the consortium partners; not all partners in the consortium are joint controllers by definition.

^iv^ The researchers are not given access to the real data but can release predefined queries on the data and will only receive back the statistical results of those queries. This is the scenario explored in the RECAP federated learning example, where Datashield software is used to obtain aggregated data.

^v^ Examples would include partners involved in project management and ethical and legal (ELSI) researchers involved in a separate work package and/or with separate deliverables. Typically, such partners perform work in relation to or inspired by (parts of) projects involving personal data processing, but these do not have a concrete role in the actual data processing.

^vi^ It should be mentioned that in many projects organisations of patient or participant representatives are partners in the project as well.
