# Supplementary material for: Joint controllers in large research consortia: a funnel model to distinguish controllers in the sense of the GDPR from other partners in the consortium
Source: Open Res Eur. 2024 Feb 9;2:80. Originally published 2022 Jun 17. [Version 2] doi: 10.12688/openreseurope.14825.2 (PMC10521071; doi:10.12688/openreseurope.14825.2)
Supplement: Peer review history

# Reviewer report: Reviewer response for version 1

**DOI:** 10.21956/openreseurope.16013.r34601

**Published:** 2023-10-09

**Authors:** Alexander Bernier

**Affiliations:** 1 McGill University, Montreal, Québec, Canada

This is a well-articulated and well-researched article on the concept of joint controllership in biomedical research consortia. 

 Overall, I wholeheartedly agree with both the legal analysis made, and the proposed model of "funnel" analysis. 

 One nuance in the literature, however, appears not to have been addressed. Namely, the authors state that the test for controllership has not changed from the DPD to the GDPR. 

 My own position is that the liability rules associated to joint controllership have changed between the DPD and the GDPR. 

 The DPD recognized a wide 'concept' of joint controller - but limited the potential liability of each controller to the acts that were associated to their responsibilities within the broader controllership arrangement. 

 According to the DPD, the concept of joint controllership was articulated in a broad manner (capturing a considerable "mix" of different institutional arrangements). However, liability arising from joint controllership was narrowly construed and associated to the activities left to that controller's oversight (see e.g. FashionID on this topic). 

 Conversely, art. 82 of the GDPR establishes the joint and several liability of each joint controller in a joint controllership arrangement (with further support at Recital 146). 

 Therefore, it would appear that there is a substantive shift in the structure of the joint and several liability rules: 

 Courts have broadly interpreted the concept in the past, but have used the structure of the liability rule to limit the arising damages. 

 The GDPR implemented a joint and several liability rule, bringing together both a "broad" legal framing (jurisprudential) and a broad potential for liability. 

 I believe that following change might benefit the article: 

 I. To describe in the review of the history of the DPD and GDPR the shift in liability rule (i.e. that the DPD had a broad conception of JCs but a narrow conception of liability (each liable only for their own acts), whereas the GDPR adopts both a broad framing for joint controllership - jurisprudentially constructed - and for liability (as constructed through statute).  

 Otherwise, however, the article is strong as stands and is recommended for indexing.

Where applicable, are recommendations and next steps explained clearly for others to follow? (Please consider whether others in the research community would be able to implement guidelines or recommendations and/or constructively engage in the debate)

Yes

Does the article adequately reference differing views and opinions?

Partly

Are all factual statements correct, and are statements and arguments made adequately supported by citations?

Partly

Is the rationale for the Open Letter provided in sufficient detail? (Please consider whether existing challenges in the field are outlined clearly and whether the purpose of the letter is explained)

Yes

Is the Open Letter written in accessible language? (Please consider whether all subject-specific terms, concepts and abbreviations are explained)

Yes

Reviewer Expertise:

Research lawyer specialised in data protection, privacy law, and the governance of biomedical research consortia.

I confirm that I have read this submission and believe that I have an appropriate level of expertise to confirm that it is of an acceptable scientific standard, however I have significant reservations, as outlined above.

# Reviewer report: Reviewer response for version 1

**DOI:** 10.21956/openreseurope.16013.r34161

**Published:** 2023-09-12

**Authors:** Javed Ahmed Shahani

**Affiliations:** 1 Sukkur IBA University, Sukkur, Pakistan

This paper addresses challenges faced by large European research consortia in managing personal data collected, generated, or shared during their collaborative research in the health sciences. The paper summarizes relevant case law, guidelines, and literature on this topic, highlighting the need for a more practical approach. The paper proposes a solution in the form of a funnel model with sequential steps to disentangle partners' responsibilities. The funnel model provides a practical framework for clarifying responsibilities within consortia, promoting smoother cooperation and GDPR compliance. Critics might argue that the funnel model introduces unnecessary complexity into an already intricate issue. The multiple sieves and criteria could lead to confusion and disputes within consortia, potentially hindering collaboration rather than simplifying controller-ship determination. Partners may argue that the model's criteria incentivize outsourcing of certain tasks, making it challenging for consortium members to engage in substantive roles without being deemed controllers. Despite the multiple sieves, grey areas persist, particularly in distinguishing between different levels of influence. Partners may question the practicality of these distinctions and their impact on data protection. In conclusion, while the funnel model offers a structured approach to resolving the joint controller-ship issue, it may face criticism for its complexity, subjectivity, and potential to create disputes

Where applicable, are recommendations and next steps explained clearly for others to follow? (Please consider whether others in the research community would be able to implement guidelines or recommendations and/or constructively engage in the debate)

Partly

Does the article adequately reference differing views and opinions?

Yes

Are all factual statements correct, and are statements and arguments made adequately supported by citations?

Yes

Is the rationale for the Open Letter provided in sufficient detail? (Please consider whether existing challenges in the field are outlined clearly and whether the purpose of the letter is explained)

Yes

Is the Open Letter written in accessible language? (Please consider whether all subject-specific terms, concepts and abbreviations are explained)

Yes

Reviewer Expertise:

GDPR, Data Protection, and Privacy in online social networks

I confirm that I have read this submission and believe that I have an appropriate level of expertise to confirm that it is of an acceptable scientific standard.

# Reviewer report: Reviewer response for version 1

**DOI:** 10.21956/openreseurope.16013.r34603

**Published:** 2023-09-08

**Authors:** Jiahong Chen

**Affiliations:** 1 The University of Sheffield, Sheffield, England, UK

The open letter addresses a practically highly important and complicated issue surrounding the identification of data controllers in the context of large research consortia. The open letter engages with the literature (including case law, regulatory guidance and scholarship) in a relevant and meaningful manner. The richer body of literature underlying the work by Becker *et al.* and Finck could have been discussed further, but for an open letter the level of engagement is sufficient as it currently stands.

 The development of the proposed funnel model is well-reasoned and struck me as practically helpful, supported with rationales both from the legal analysis as well as the inputs from the webinar. However, at certain points, especially when explaining the four sieves with regard to the interpretations of (joint) controllership in the literature, it is not entirely clear whether the authors' own interpretation is one that one would reasonably come to in the light of the relevant case law and regulatory guidance (what the definition *is* - doctrinally), or one that a future interpreter (whether a court or an enforcer) is expected to rightly take (what the definition *should be* - theoretically). I appreciate the two questions are not always distinguishable, not least considering the legal uncertainties in this area, but it would have been more helpful if the open letter made it slightly clearer which of the assumptions are those on which the stakeholders can rely somewhat safely, and which would require further advocacy for a particular interpretative approach.

 The open letter would also benefit from taking a more critical approach towards the current governance models of research consortia rather than taking current practices at face value. This is particularly the case when it comes to sieving out stakeholders who exercise their influence in a way that the level and nature of such influence is uncertain due to how they choose to interact with other actors. By way of analogy, the shareholders of a company are (usually) not held responsible as data controllers for decision-making on data uses by that company, even if they have exercised influence through the corporate governance structure. That is because through the establishment of a legal entity (which involves individual shareholders ceding some of their rights), the responsibilities have been inextricably bundled. The fact that some large research initiatives have taken the form of a research consortium as described by the open letter is an *organisational choice* that comes with certain legal implications. Of course, if this is a choice that brings forth particular benefits, then there is scope for the law to facilitate this governance structure by, say, defining the roles, rights and duties more clearly, but equally there is scope to question whether chosen way the collaborate is indeed the best structure within the current legal framework. I appreciate this is an open letter and perhaps more practically oriented, but this should be highlighted as a question for future research.

 Overall, the open letter is well-researched and well-written with a clear, objective and accessible writing style, concluding with a largely clear and plausible recommended approach.

 Specific minor comments:

1. At a few points the use of language in the open letter comes across as being slightly too colloquial for academic writing (e.g. "This would take things to the extreme.").
2. "The consensus was that a large number of joint controllers does add any value to the data subjects/participants in the research of the consortium." - I am unsure I follow this sentence.

Where applicable, are recommendations and next steps explained clearly for others to follow? (Please consider whether others in the research community would be able to implement guidelines or recommendations and/or constructively engage in the debate)

Partly

Does the article adequately reference differing views and opinions?

Yes

Are all factual statements correct, and are statements and arguments made adequately supported by citations?

Yes

Is the rationale for the Open Letter provided in sufficient detail? (Please consider whether existing challenges in the field are outlined clearly and whether the purpose of the letter is explained)

Yes

Is the Open Letter written in accessible language? (Please consider whether all subject-specific terms, concepts and abbreviations are explained)

Yes

Reviewer Expertise:

Data protection law, cybersecurity law, law and AI, data ethics, internet regulation

I confirm that I have read this submission and believe that I have an appropriate level of expertise to confirm that it is of an acceptable scientific standard.

# Reviewer report: Reviewer response for version 1

**DOI:** 10.21956/openreseurope.16013.r29580

**Published:** 2022-09-02

**Authors:** Anne Bahr

**Affiliations:** 1 Sanofi, Paris, France

This article aims at providing practical criteria for defining which parties within a large consortium (especially in the domain of scientific research) are joint-controllers and which one are not.

 Its analysis is based on most important EU-level documents and guidelines as well as key EU-level judgments and other publications. These documents are very well understood and exposed by the authors, in language accessible to persons who knows nothing about personal data protection legal framework.

 The authors are proposing a smart 4-steps approach assimilated to a ‘funnel-and-sieves’ model to distinguish partners which are really joint-controller from those which should not be considered as such, despite the interpretations some can make from the CJEU decisions and the EDPB opinion papers.

 This approach seems very good and pragmatic to me.

- However, it lacks some clarity, especially for the first sieve when saying that “ *Controllers are only those who **are in charge** of the specific studies which result from the project, or, in the case of a data platform, who **control the studies which are performed** via that platform*”. Being “in charge” and “controlling” may be interpreted very differently by scientific with different backgrounds and deserves more clarity. From an industrial background, those who are “in charge” of a study are the service providers/ CROs, whereas the industry is the Sponsor, and therefore the controller. My company as well as out leading authority (the Cnil) would consider that investigational sites/ hospitals are “in charge of the specific studies” as processors for the study as well as being independent controllers for the management of the health data in their premises. This case is still under debate between Member States Supervisory Authorities and will not be addressed here. However, the authors should consider this misalignment for defining the first/ most important sieve criteria.
- The criteria for the second sieve are clear and seem very interesting to me: “ *Those partners who provide services which also could have been subcontracted but for any variety of reasons are performed by consortium members, are usually not controllers.*”
- The criteria for the next two sieves also seems clear and good to me. However, with the necessary split between the situations a and b, a schema or a figure would make it easier to follow. This is why I ticked “partly” to the question “Where applicable, are recommendations and next steps explained clearly for others to follow”.
- Additionally in the 3 ^rd^ sieve (3a), when referring to partners who “ *perform the analyses*”, some may read that this is a task of a processor.

 

 In conclusion, this is a very interesting well written article which, with the few changes proposed, will be very helpful for scientists to better define roles in complex consortium (as well as in simple ones) and therefore also improve the information to be provided to patients.

 I therefore suggest the following corrections:

  

1. The following text is not very clear with regards to who is who. I suggest using the same term (either the name of the body (Wirtschaftsakademie) or the role (fan page administrators) and add terms such as “only” or “also” to make the reading easier.
*It held that **there are two controllers in relation to a Facebook fan page**. First, **Facebook serves as a controller** from the perspective of personal data protection, as it primarily determines the purposes and means for users and visitors of fan pages. Secondly, **fan page administrators serve as controllers** as they subscribe to Facebook’s conditions of use. Fan page administrators were considered to determine the purposes (the objective of establishing a fan page) and the means of data processing by defining parameters of data collection, including the target audience.* ***Considering that a fan page administrator influences these modalities (carried out by Facebook), the Wirtschaftsakademie was classified by the Court as a controller, even though it only received analytics data in anonymized form. The Court thereby ruled in favour of a broad interpretation of controllership*** *.*
2. The following text is not very clear with regards to the reference and the author (i.e., not clear who/ what is the reference for “Finck” as it is the first time it is cited). I suggest moving the reference from term “above” to the term “Finck” for better understanding that the reference applies to the author and not to the case law.

*From a very different perspective Finck criticises the broad scope of joint controllership in the case law discussed above.*

1. The term “NOT” is missing in this sentence I think
*The consensus was that a large number of joint controllers does **NOT** add any value to the data subjects/participants in the research of the consortium.*
2. As explained above, the following text is not very clear (especially the terms “in charge” and “control the studies”) and deserves some clarification or examples.
  “ *Although the project proposal and the DoA describe purposes and means in broad lines, these ambitions are refined during the project when performing actual studies involving data processing. Controllers are only those who **are in charge** of the specific studies which result from the project, or, in the case of a data platform, who **control the studies which are performed** via that platform*”
3. As explained above, the 3a sieve is not very clear. I suggest the following bold modification or an equivalent one “ *3a. The partner(s) have drafted the protocol and/or **deciding on how to** perform the analyses on which the results will be based and/or*”

Where applicable, are recommendations and next steps explained clearly for others to follow? (Please consider whether others in the research community would be able to implement guidelines or recommendations and/or constructively engage in the debate)

Partly

Does the article adequately reference differing views and opinions?

Yes

Are all factual statements correct, and are statements and arguments made adequately supported by citations?

Yes

Is the rationale for the Open Letter provided in sufficient detail? (Please consider whether existing challenges in the field are outlined clearly and whether the purpose of the letter is explained)

Yes

Is the Open Letter written in accessible language? (Please consider whether all subject-specific terms, concepts and abbreviations are explained)

Yes

Reviewer Expertise:

Privacy & Data Protection

I confirm that I have read this submission and believe that I have an appropriate level of expertise to confirm that it is of an acceptable scientific standard.
